# Anticoagulation may contribute to antimicrobial treatment of Lemierre syndrome: a case report

**DOI:** 10.1186/s12959-021-00336-0

**Published:** 2021-11-04

**Authors:** Jie Ge, Peipei Zhou, Yifei Yang, Tianshu Xu, Xu Yang

**Affiliations:** Department of stomatology, Changzhou First People’s Hospital, Juqian Street, Changzhou, 185 Jiangsu China

**Keywords:** Anticoagulation, Infectious disease, Thromboembolism, Lemierre syndrome, Case report

## Abstract

**Background:**

Lemierre syndrome (LS) is characterized by multisystemic infection beginning in the oropharynx, local thrombophlebitis (typically, of the internal jugular vein) and peripheral embolism. No evidence-based guidelines exist for the management of this disease, and the use of anticoagulation therapy remains particularly controversial.

**Case presentation:**

A 61-year-old man presenting with left neck swelling, odynophagia, and dyspnea underwent emergency surgery and received intravenous antibiotics. The primary infection was controlled on hospital day 5, but on day 6 sudden leukocytosis and hypoxemia were observed. CT angiography revealed an intraluminal filling defect in the pulmonary artery on day 8. LS was diagnosed and anticoagulation therapy was initiated. The WBC count, which had maintained its peak values in the previous 2 days, decreased instantly after initiation, and follow-up controls showed thrombus resolution.

**Conclusions:**

Our case supports the notion that anticoagulation therapy may be a valid supplement to antimicrobial therapy in LS, especially in the presence of a possibly young thrombus as suggested by clinical worsening.

## Background

Despite its nonnegligible annual incidence of > 1/100000, Lemierre syndrome (LS) often goes undiagnosed due to its non-specific symptoms [1, 2]. The most accurate definition of LS should include 1) infection that originates in the oropharynx, mastoiditis, oral cavity and sinusitis, 2) isolation of *Fusobacterium necrophorum* and 3) local thrombophlebitis, typically of the internal jugular vein, producing disseminated emboli to the periphery (often septic pulmonary emboli) [2–4]. However, as an obligate anaerobic bacterium, *Fusobacterium necrophorum* is often difficult to isolate in blood cultures. It requires a longer incubation period than other bacteria, and cultures can be false negative if antibiotics are administered before sample collection. In addition, other bacteria may cause the same clinical syndrome. Therefore, a clinical diagnosis of LS is considered to be still valid even when *Fusobacterium necrophorum* is undetected or other bacteria are detected [5–7]. Delayed treatment of LS can lead to multisystemic organ dysfunction, potential end-stage organ damage or death. Systemic emboli and septic pulmonary emboli can also be life threatening. Therefore, early diagnosis and treatment of LS are vital. The key tools of physicians are antibiotic therapy, anticoagulation therapy and drainage of abscesses [2]. The use of anticoagulation therapy remains controversial, and it is unclear whether anticoagulation treatment improves patient outcomes. Here, we report a case in which anticoagulation initiated after clinical worsening was followed by prompt clinical and laboratory improvement, supporting a role of anticoagulation in the treatment of LS.

## Case presentation

**Fig. 1:**
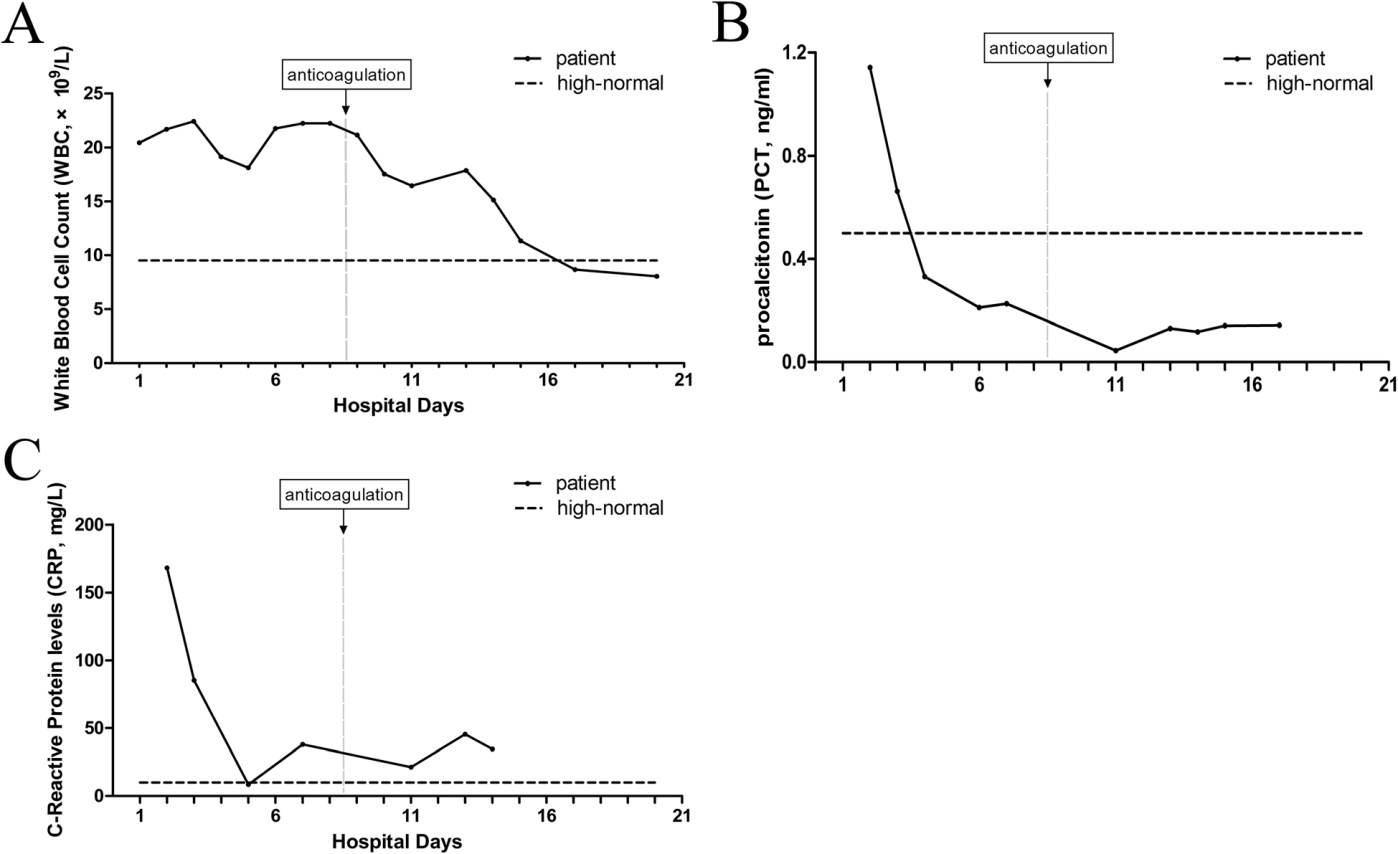
Blood investigations. **A**: WBC count. **B**: PCT level. **C**: CRP level

**Fig. 2:**
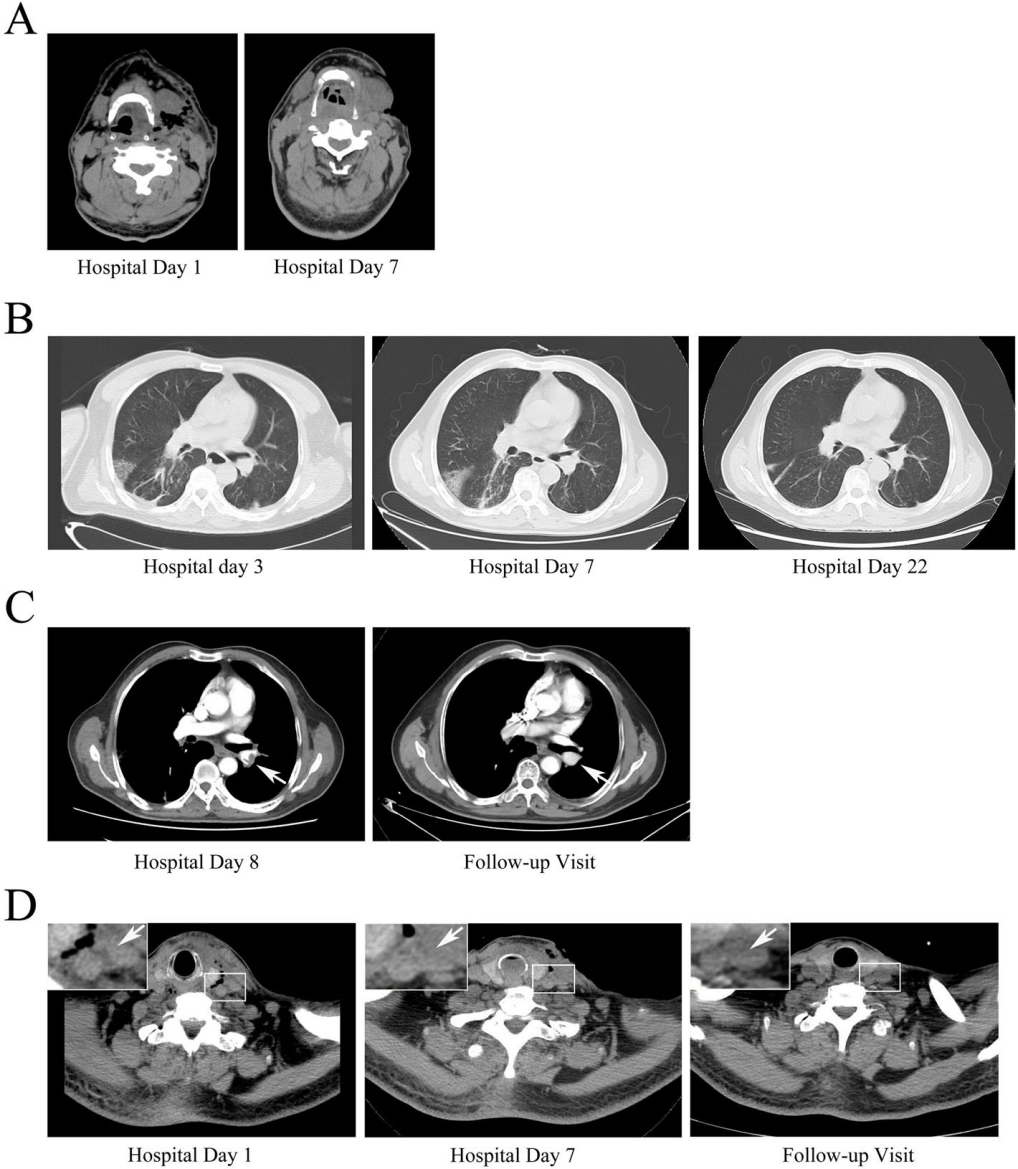
Imaging. **A**: Neck CT. **B**: Chest CT. **C**: Pulmonary artery CT. White arrow: pulmonary emboli in the “Hospital Day 8” image; pulmonary emboli disappeared in the “Follow-up Visit” image. **D**: Internal jugular vein CT. White arrow: signs of inflammation next to the internal jugular vein in “Hospital Day 1” and “Hospital Day 7” images; the signs of inflammation disappeared in the “Follow-up Visit” image

A 61-year-old man presented to the oral and maxillofacial surgery service with a 5-day history of left neck swelling, odynophagia, and dyspnea without chest pain or cough. He reported a history of 7-day odontalgia and 3-day intravenous cefotiam (1 g every 12 h) and metronidazole (500 mg every 8 h) treatment. He was otherwise healthy, with no known allergies. His weight was 65 kg. At the time of admission, the patient had an oxygen saturation (SpO) of 98% while breathing ambient air. Laboratory data revealed a white blood cell count (WBC) of 20.45 × 10^9^ /L (Fig. 1 A), procalcitonin (PCT) of 1.142 ng/mL (Fig. 1 B), C-reactive protein (CRP) of 168.23 mg/L (Fig. 1 C), random blood glucose of 16.02 mmol/L and glycosylated hemoglobin of 8.7%. Computed tomography (CT) of the neck revealed diffusive swelling combined with pneumatosis (Fig. 2 A). The patient was then diagnosed with cervical necrotizing fasciitis.

He underwent emergency incision and drainage surgery and received empirical treatment with intravenous imipenem-cilastatin sodium (1 g every 8 h) and metronidazole (500 mg every 8 h). His blood glucose was controlled by insulin. Anaerobic cultures of pyogenic fluids and blood were obtained on admission and were sterile 5 days later, which may have been due to the antimicrobial treatment before admission. After five days of antimicrobial treatment (hospital day 5), the patient’s WBC count had gradually decreased to 18.12 × 10^9^ /L (Fig. 1 A; hospital day 5) and CRP to 8.51 mg/L (Fig. 1 C; hospital day 5). However, he had an SpO of 80% in room air and 96% with oxygen inhalation (5 L/min) on the sixth hospital day. Laboratory data showed that his WBC count increased to 21.77 × 10^9^ /L (Fig. 1 A; hospital day 6), while his PCT level decreased to 0.211 ng/mL (Fig. 1 B; hospital day 6). A repeat CT scan revealed that the neck infection had regressed (Fig. 2 A) and that the pulmonary infection was stable (Fig. 2 B). Chest CT angiography revealed an intraluminal filling defect in the lower branch of left pulmonary artery on the eighth hospital day (Fig. 2 C). This manifestation was considered consistent with septic pulmonary embolism derived from the internal jugular vein, which was very close to the infection focus (Fig. 2 D). LS was diagnosed.

Anticoagulation therapy was performed with low molecular weight heparin (4250 U every 12 h) for the following 4 days. His WBC gradually decreased to 17.87 × 10^9^ /L (Fig. 1 A; hospital day 13). Low molecular weight heparin was replaced with warfarin (3 mg every day) on hospital day 12. The international normalized ratio (INR) was monitored constantly and controlled between 2 and 2.5. The patient’s WBC count decreased to 8.67 × 10^9^ /L on hospital day 17 and remained normal on the following days (Fig. 1 A). Repeat chest CT showed that the pulmonary infection was cured on hospital day 22 (Fig. 2 B). He was discharged from the hospital on hospital day 24 and continued anticoagulation therapy. When he returned for a follow-up visit at the twentieth week, the embolus had dissolved (Fig. 2 C), and anticoagulation therapy was discontinued. No haemorrhage occurred during anticoagulation therapy.

## Discussion and conclusions

In this case, the primary infection was controlled on hospital day 5, as proven by CT evaluation and decreased WBC and PCT levels. The sudden increase in WBC count and decrease in SpO on hospital day 6 were considered to be caused by newly formed septic pulmonary emboli. Pulmonary emboli above the subsegmental level are a possible, but rare complication of LS, and 3 days were required for diagnosis of LS. As a result, anticoagulation therapy was not initiated until hospital day 8. The WBC count maintained its peak value on hospital days 6–8 before anticoagulation therapy was initiated, but rapidly decreased after initiation. This quick response suggests that anticoagulation therapy may have contributed to the infection resolution achieved by antimicrobials.

In a review of 137 cases of LS, the authors found that 4–6 weeks of carbapenem or piperacillin/tazobactam combined with metronidazole were effective in terms of infection control, but the reason for the choice of such a duration was not explained in any of the included papers [6]. Our antimicrobial treatment was maintained until the WBC counts remained normal for 1 week and clinical symptoms improved. The duration was 24 days in total and 16 days after anticoagulation therapy proceeded, which is much shorter than the average. However, it is still unknown whether anticoagulation therapy is essential, which is an important issue especially in cases, such as the one we described, that are complicated by large intraluminal emboli.

In the past few decades, evidence remained poor regarding the management of LS, particularly regarding its potentially life-threatening thromboembolic complications. Consequently, physicians must make their decisions based on small case series or anecdotal cases when facing uncommon thromboembolic conditions, such as LS. Physicians who are opposed to anticoagulant treatment confirm that the thrombus is caused by an infection process and will be resolved at the same time when the infection is resolved. In contrast, some authors confirm that anticoagulant therapy may reduce the morbidity and mortality of serious complications, such as cavernous sinus thrombosis or pulmonary embolism. Theoretically, a septic thrombus sequesters bacteria and creates a barrier to antibiotic penetration. When the thrombus is dissolved by anticoagulants, the bacteria are exposed to a higher concentration of antibiotics, increasing accessibility [8]. Our case supports this hypothesis precisely. Unfortunately, randomized controlled trials are impractical to investigate the use of anticoagulation therapy in the treatment of LS due to the rarity of this illness. Based on small case series, the proposal has been advanced to reserve this approach to bilateral disease only; a single-center retrospective series has found no association with thrombus recanalization, although it is unclear whether recanalization is clinically relevant [9]. The improvement seen in our case despite the absence of bilateral disease and the finding of recanalization following anticoagulation could be explained by the likelihood that the thrombus had developed recently, as suggested by the sudden increase in infection parameters, and may have been more vulnerable to anticoagulation than an older, organized thrombus. This possibility is consistent with the known biology of venous thrombi [10] and should be explored by future research.

A recent European collaborative study performed an individual patient-level analysis of 712 cases published globally from 2000 to 2017 [1]. The authors could not find disease-specific elements against the safety of anticoagulation and drew the conclusion that therapeutic anticoagulation is indicated for LS if there are no contraindications. Nevertheless, the authors did not provide definitive guidelines for the duration of anticoagulation. According to the American College of Chest Physicians guidelines for provoked thrombotic events, anticoagulation therapy is recommended for a duration of at least 3 months [11], which may be available for reference to Lemierre syndrome presented pulmonary embolism. Our patient received a treatment duration of 20 weeks because his follow-up visit was delayed by the outbreak of COVID-19. A limitation of our case is the lack of microbial isolation. However, the diagnosis of LS is currently accepted even without microbial isolation in the presence of a clinical diagnosis of sepsis from an oropharyngeal focus and thrombi or emboli consistent with the primary infection location [5]. This case satisfies the broad criteria of Lemierre syndrome, that are currently accepted, but not the restrictive or classic criteria that require evidence of jugular vein thrombosis and *Fusobacterium necrophorum*, and that future management studies should report results for both subpopulations.

In conclusion, therapeutic anticoagulation may be considered in the management of LS, while further research is needed to evaluate whether the use of anticoagulant and antibiotics leads to better clinical outcomes than the use of antibiotics alone.

### Patient perspective

I’m the daughter of the patient. We are grateful to the doctors’ help in Changzhou First People’s Hospital and happy to share the treatment experience of my father.

## Data Availability

The datasets generated and/or analysed during the current study are not publicly available due to patient privacy protection but are available from the corresponding author on reasonable request.

## Abbreviations

LS: Lemierre syndrome
SpO: Oxygen saturation
WBC: White blood cell count
CRP: C-Reactive protein
PCT: Procalcitonin
CT: Computed tomography
INR: International normalized ratio
